# Nécrose digitale révélant une vascularite rhumatoïde

**DOI:** 10.11604/pamj.2014.18.243.5028

**Published:** 2014-07-25

**Authors:** Faida Ajili, Nadia Ben Abdelhafidh

**Affiliations:** 1 Service de Médecine Interne, Hôpital Militaire de Tunis, Tunisie

**Keywords:** Nécrose digitale, vascularite rhumatoïde, doigt, digital necrosis, rheumatoid vasculitis, finger

## Image en medicine

La vascularite rhumatoïde constitue une manifestation rare < 1% et grave de la PR. Il s'agit d'une vascularite nécrosante des vaisseaux de petit calibre. Les signes habituels sont une vascularite cutanée et une multinévrite. Nous rapportons une nouvelle observation. Patient âgé de 69 ans, tabagique à 50 PA sans antécédents particuliers, consulte pour une polyarthrite touchant les petites et grosses articulations évoluant depuis 2 mois associée à une nécrose digitale des 2 mains (2^ème^, 3^ème^, 4^ème^ et 5^ème^doigt) et à des douleurs d'allures neuropathiques aux membres inférieurs. A l'examen le patient était apyrétique, TA = 130/70 cmHg, Pouls perçus à 80/mn. On notait une synovite des 2 poignets et une arthrite des 2 genoux et chevilles. Sur le plan cutané, il avait une nécrose digitale sèche touchant les 2^ème^, 3^ème^, 4^ème^ et 5^ème^ doigts des 2 mains. La biologie montrait un syndrome inflammatoire biologique. La NFS, les bilans rénal, hépatique, CPK-LDH et phosphocalcique étaient normaux et la protéinurie de 24 h négative. Le bilan immunologique (ANCA, AAN) était négatif et les Ac anti CCP++ > 200U/ml. Plusieurs diagnostics ont été évoqués mais éliminés en particulier les vascularites nécrosantes. Finalement, une polyarthrite rhumatoïde associée à une vascularite a été retenue devant 6 critères de l'ACR. Le patient était mis sous corticothérapie 1mg/kg/j associée à des cures mensuelles de cyclophosphamide 700mg/m^2^ de surface corporelle (x 6 cures). L’évolution était marquée par une amélioration de l’état clinique, une reprise de l'appétit, une disparition des arthrites et des lésions de purpura et une stabilisation des lésions nécrotiques. Le recul est de 6 mois.

**Figure 1:**
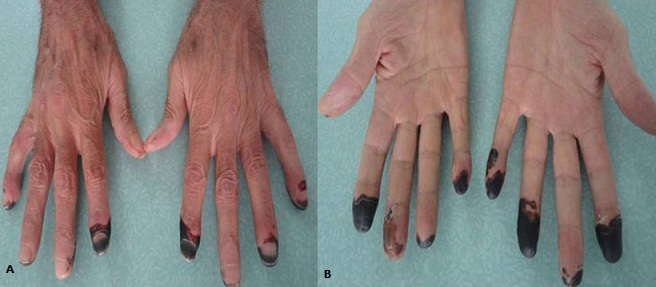
Nécrose digitale sèche touchant les 2ème, 3ème, 4ème et 5ème doigts des 2 mains chez notre patient

